# Postponing tumor onset and tumor progression can be achieved by alteration of local tumor immunity

**DOI:** 10.1186/s12935-021-01765-7

**Published:** 2021-02-10

**Authors:** Yan Mei, Mingdian Wang, Guanming Lu, Jiangchao Li, Lixia Peng, Yanhong Lang, Mingming Yang, Lingbi Jiang, Changzhi Li, Lisheng Zheng, Zhijie Liu, Dehuan Xie, Lingling Guo, Bijun Huang, Musheng Zeng, Yanxia Shi, Chaonan Qian

**Affiliations:** 1 State Key Laboratory of Oncology in South China, Collaborative Innovation Center for Cancer Medicine, Sun Yat-Sen University Cancer Center, 651 Dongfeng East Road, Guangzhou, 510060 Guangdong China; 2 Department of Breast and Thyroid Surgery, Affiliated Hospital of Youjiang Medical University for Nationalities, Basie, 533000 China; 3 Vascular Biology Research Institute, School of Basic Course, Guangdong Pharmaceutical University, Guangzhou, 510006 China; 4 Department of Medical Oncology, Sun Yat-Sen University Cancer Center, Guangzhou, 510060 China; 5 Department of Nasopharyngeal Carcinoma, Sun Yat-Sen University Cancer Center, Guangzhou, 510060 China

**Keywords:** Breast cancer, PyMT mouse model, Dendritic cells, IL-6, Angiogenesis, VEGF

## Abstract

**Background:**

It has been known for years that the same genetic defects drive breast cancer formation, yet, the onset of breast cancer in different individuals among the same population differs greatly in their life spans with unknown mechanisms.

**Methods:**

We used a MMTV-PyMT mouse model with different genetic backgrounds (FVB/NJ vs. C57BL/6J) to generate different cancer onset phenotypes, then profiled and analyzed the gene expression of three tumor stages in both Fvb.B6 and Fvb mice to explore the underlying mechanisms.

**Results:**

We found that in contrast with the FVB/N-Tg (MMTV-PyMT) 634Mul mice (Fvb mice), mammary tumor initiation was significantly delayed and tumor progression was significantly suppressed in the Fvb.B6 mice (generated by crossing FVB/NJ with C57BL/6J mice). Transcriptome sequencing and analysis revealed that the differentially expressed genes were enriched in immune-related pathways. Flow cytometry analysis showed a higher proportion of matured dendritic cells in the Fvb.B6 mice. The plasma levels of interleukin-6 (IL-6) and vascular endothelial growth factor (VEGF) were significantly reduced in the Fvb.B6 mice. IL-6 also impaired the maturation of bone marrow dendritic cells (BMDCs) of the Fvb mice in vitro.

**Conclusion:**

All these findings suggest that immunity levels (characterized by a reduced IL-6 level and intact DC maturation in Fvb.B6 mice) are the key factors affecting tumor onset in a murine mammary cancer model.

## Background

Breast cancer is the most common type of malignancy for women globally [1]. With 1.67 million new cases per year, it is the 5th ranked cause of death [2]. It is therefore, crucial to determine the underlying mechanisms for its tumorigenesis and cancer progression. Most solid tumors occur in middle aged patients, although, affected individuals might be susceptible to cancer from birth. We previously hypothesized that there is a mechanism in the human body that can postpone the onset of cancer [3], which may be related to host immune functions. Accumulating evidence has shown that immunity is of vital importance in breast cancer initiation and progression [4, 5], and that high levels of protective immunity can efficiently suppress tumor growth [6, 7]. The Fvb (FVB/NJ) mouse strain is susceptible to mammary tumors, while the C57BL/6J strain is more resistant to inflammation-induced mammary tumors [8]. One possible reason for this is that the major histocompatibility complex (MHC)-related immunity pathway mediates resistance associated with the H-2b haplotype in the C57BL strain [9, 10].

Dendritic cells (DCs) are professional antigen presenting cells (APCs), and they display antigen complexed with major histocompatibility complexes (MHCs) on their surfaces. Previous studies have shown that tumor-derived soluble mediators, e.g., IL-6, TGF-β, and CCL2 can significantly suppress DC’s functions and impair their ability to initiate antitumor immune responses [11]. Accumulation of IL-6 in the tumor microenvironment is associated with a functional defect in the DCs of cancer patients [12].This inhibitory effect is believed to be controlled by the IL-6-triggered activation of JAK2/STAT3 signaling in the DCs [13]. In addition, vascular endothelial growth factor (VEGF) is a critical factor that can inhibit the migratory ability and immune function of mature dendritic cells [14]. Moreover, the VEGFR antagonist can impair dendritic cell maturation [15].

Mouse models are useful for studying the molecular mechanisms of breast cancer [16, 17], with one of the most studied models being the FVB/N-Tg (Mouse Mammary Tumor Virus Polyoma Middle T Antigen, MMTV-PyMT) 634Mul model, in which the middle T-antigen of the polyoma virus is expressed as an oncogene in mammary tissues thereby driving tumor formation [18].

In the present study, Fvb.B6 F1 hybrids were generated by crossing FVB/N-Tg (MMTV-PyMT) 634Mul mice with C57BL/6J mice. In doing so, mammary tumor initiation, growth, and metastasis were suppressed in the Fvb.B6 F1 hybrids, supporting our hypothesis of postponed cancer onset [3]. Using the MMTV-PyMT mouse model, we sequenced and analyzed the global expression profiles of mammary tumors from the FVB/NJ background and F1 progeny from crossing them with C57BL/6J mice. By comparatively analyzing the global expression profiles of mammary tumors from adenoma/mammary intraepithelial neoplasia (MIN), late carcinoma, and lung metastasis, we identified antigen-presenting related genes and pathways involved in tumor progression and metastasis. We also found that Fvb.B6 mice had more mature DCs compared with Fvb mice, whose maturation may be suppressed by IL-6.

## Materials and methods

### Mice and animal care

FVB/N-Tg(MMTV-PyMT)634^Mul^ mice with an FVB/NJ (Fvb) background were purchased from the Jackson Laboratory (Bar Harbor, ME, USA). C57BL/6J (B6) mice were purchased from the Guangdong Medical Laboratory Animal Center (Foshan, China). Mice were housed under specific pathogen-free conditions in the Animal Center of Guangdong Pharmaceutical University.

FVB/N-Tg(MMTV-PyMT)634^Mul^ male mice were crossed with C57BL/6J females to produce a transgene-positive Fvb.B6 F1 hybrid female progeny (Additional file 1: Fig. S1A). These virgin transgene-positive F1 hybrids and FVB/N-Tg(MMTV-PyMT)634^Mul^ mice were euthanized for tissue harvesting. Mammary tumors were harvested when the diameter of the largest tumor per mouse was between 2 and 10 mm, which corresponded to the Adenoma/MIN and late carcinoma stages, respectively (Additional file 1: Fig. S1B). Lung metastases were harvested at the 13 weeks old for the Fvb mice and 16 weeks for the F1 hybrids.

### RNA-sequencing and analysis

Nine tumor samples from the FVB/N-Tg(MMTV-PyMT)634^Mul^ and Fvb.B6 F1 hybrids were placed in RNA storage reagent (TIANGEN) for RNA isolation. Total RNA was isolated from the 18 samples, with three tumor samples per group. RNA quality was assessed using an Agilent 2100 Bioanalyzer (Agilent Technologies). The RNA was sheared and reverse transcribed using random primers to obtain the cDNA for library construction. The library was sequenced through the BGISEQ-500 platform at the Beijing Genomic Institution (Wuhan, China).

All statistical analyses were performed by using R v3.3.1. After removing transcriptionally inactive genes (read counts per million were < 1), the high-confidence gene counts were obtained. For gene expression analysis, the matched reads were calculated and normalized to RPKM using RESM software [19]. The R package edgeR v3.18.1 was used to perform statistical analyses on the gene counts and to detect differentially expressed genes (DEGs). DEGs at each stage were used for further analyses of GO (gene ontology), biological processes and KEGG (Kyoto Encyclopedia of Genes and Genomes) pathways using the R package clusterProfiler v3.4.4 [20]. The heatmap of the immune-related differentially expressed genes was constructed using the R package pheatmap v1.0.8.

### RT-PCR

The RNA was converted to cDNA using the PrimeScript™ II 1st Strand cDNA Synthesis Kit (Takara), followed by reactions using the Roche LightCycler 480 System (Roche). Each 10 μl reaction included 5 μl of ChamQ SYBR qPCR Master Mix (Vazyme), 0.8 μl of RNAse-free water, 4 μl of cDNA (5 nM) and 0.2 μl of primer. Actb was used as the internal control gene to determine the relative fold change using the 2^−ΔΔCt^ method. The qPCR primers were obtained from PrimerBank.

### Histological examination

Murine lungs and mammary tumor tissues were fixed, embedded in paraffin, sectioned at 3-μm and stained with hematoxylin and eosin (H&E). Whole-mount carmine alum staining was performed according to the standard protocol [21]. Briefly, pairs of inguinal mammary glands were excised and flattened on a microscope slide, followed by air drying for 5 min, fixated overnight in Carnoy’s solution (75% absolute ethanol and 25% glacial acetic acid [Sigma-Aldrich]), then washed in 70% ethanol for 30 min, rinsed in distilled water for 30 min, and stained in carmine alum (Sigma-Aldrich) for two days or longer. Slides were dehydrated in three concentrations of ethanol (70%, 95%, and 100%) for 30 min at each step. Dehydrated mammary glands were defatted in toluene for at least 2 days. Slides were mounted with cover-slips using Permount mounting media, and whole mounts were digitally photographed on a stereomicroscope. To examine the lung metastasis foci, tissues were fixed in Bouin’s solution for two days. Metastatic lesions larger than 0.5 mm in diameter on the surface of the major organs were counted macroscopically.

### Immunohistochemistry staining

Three-micron paraffin-embedded mammary tumor sections were used for immunohistochemical (IHC) staining. After being deparaffinized and rehydrated, the tissue sections were incubated with anti-Ki67 (Cat#ab15580, Abcam), IL-6 (Cat# ab208113, Abcam), and CD31 (Cat# 11265-1-AP, Proteintech) antibodies overnight at 4 °C. A primary antibody binding to the tissues was detected using a HRP-conjugated secondary antibody (Cat#ZB-2306, ZSGB-BIO). All sections were stained with DAB and counterstained with hematoxylin. Images were taken in a 400 × field, quantified using IPP 6.0 software and assessed by two experimenters.

### Flow cytometry analysis

Tumor tissues were weighed, cut into small fragments (< 1 mm), and digested in 15 mL of dissociation solution (RPMI 1640 medium (Gibco, 11875-093) supplemented with 10% FBS, Collagenase type I (200 U/mL, Sigma, C0130-100MG) and DNase I (100 mg/mL, Roche, 10104159001)) for 1 h at 37 °C. Erythrocytes were lysed using a red blood cell lysis buffer (BD Bioscience, 555899). Cell suspensions were passed through 70-mm cell strainers (Fisherbrand, 22363548), and then washed and resuspended in a staining buffer (PBS with 2% FBS).

Dendritic cell expression markers were determined by FACS analyses after surface staining with anti-mouse specific antibodies conjugated with PE, FITC, allophycocyanin, PerCP-cy5.5 or PE-cy7. These antibodies included anti-MHC Class II (Cat#86212-80-100), anti-CD11c (03212-50-100), anti-CD80 (02912-60-100) and anti-CD86 (08912-77-100), all were purchased from BioGems. All stained cells were analyzed on a CytoFLEX Flow Cytometer (Beckman Coulter), and the data analyzed using CytExpert software v2.0.

### Cytokine analysis

Multiplex analysis of 32 cytokines using the 96-well Milliplex MAP mouse cytokine/chemokine magnetic bead panel (MCYTOMAG-70K-32, Millipore, MA) was performed according to the manufacturer’s instructions. The panel included the following cytokines: granulocyte colony stimulating factor (G-CSF), Eotaxin, granulocyte–macrophage colony-stimulating factor (GM-CSF), M-CSF, IFN-g, IL-1a, IL-1b, IL-2, IL-3, IL-4, IL-5,IL-6, IL-7, IL-9, IL-10, IL-12(p40), IL-12(p70), leukemia inhibitory factor (LIF), IL-13, CXCL5, CXCL9 IL-15, IL-17, interferon g-induced protein-10 (IP-10), murine keratinocyte chemoattractant (MKC), monocyte chemoattractant protein-1 (MCP-1), macrophage inflammatory protein (MIP)-1a, MIP-1b, MIP-2, regulated on activation, normal T cell expressed and secreted (RANTES), VEGF, and TNF-a.

### Isolation of bone marrow cells and generation of BMDCs

Bone marrow was harvested from FVB/NJ female mice (N = 5) and red blood cells lysed with a lysis buffer. Bone marrow cells were plated at 1 × 10^6^ per mL in a basal medium containing mouse GM-CSF (1X) and mouse IL-4(1X), and bone marrow derived dendritic cells were induced by TNF-α according to the manufacturer’s instructions (MultiSciences, Cat# CK20101). On day 6, mouse IL-6 10 ng/mL (Peprotech, Cat#216-16) was added.

### Statistical analysis

Each experiment was performed at least three times, and analyses were performed by using GraphPad Prism. The data are presented as mean ± standard error. Student's t-test or ANOVA were used to compare the differences between two groups or among more groups. P values were used to denote statistical significance. Levels of significance were *p ≤ 0.05, **p ≤ 0.01, and ***p ≤ 0.001.

## Results

### PyMT-induced mammary tumor initiation is delayed in F1 hybrids with significant survival benefits for the hosts

**Fig. 1:**
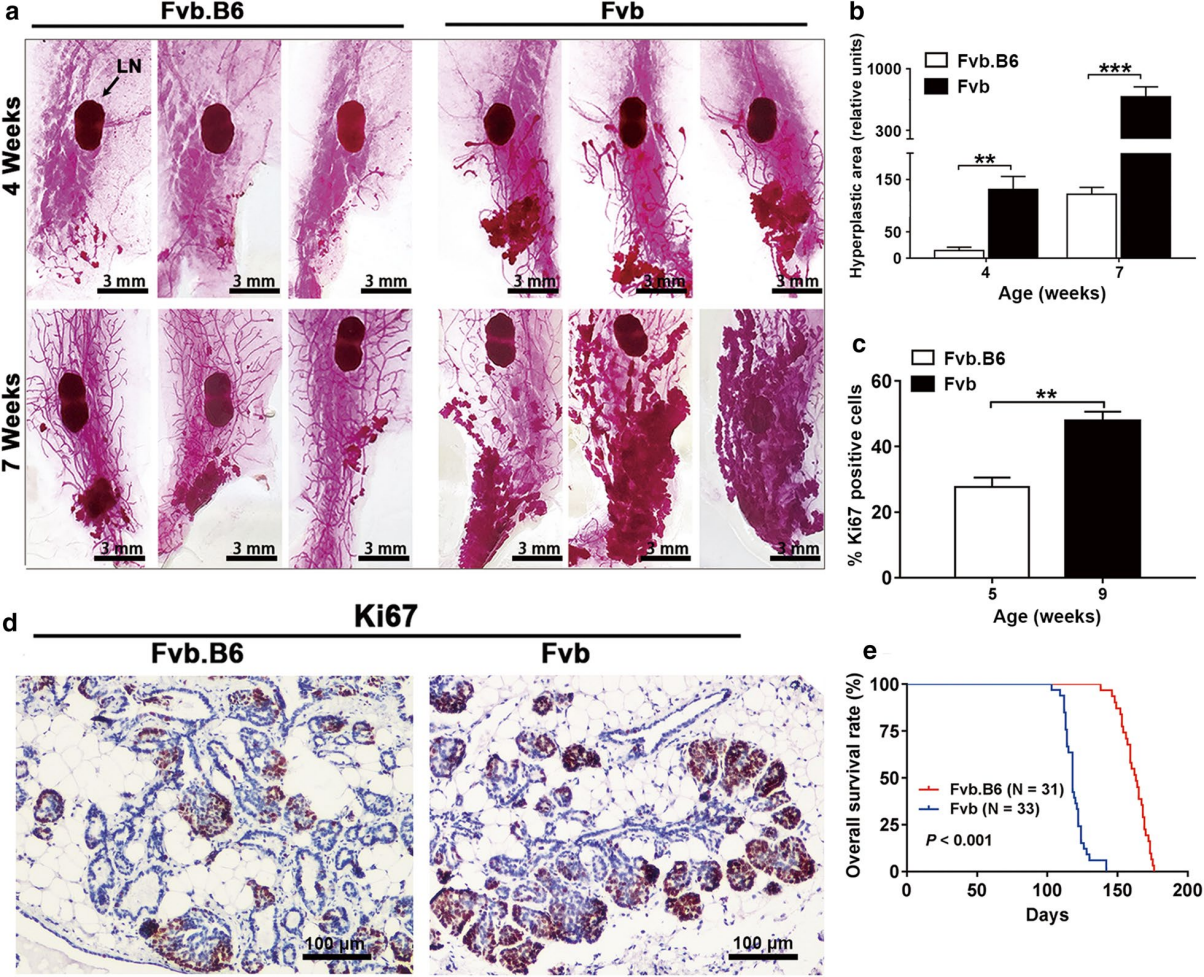
Mammary tumor initiation is delayed by a B6 genetic background, resulting in prolonged overall survival. **a** Whole mounts of #4 mammary glands from Fvb.B6 (n = 6) and Fvb (n = 6) mice at 4 and 7 weeks old. LN = lymph node. **b** The total area of hyperplastic foci in whole mount mammary glands was measured using Image J. The data represents the mean area of hyperplasia ± SEM from Fvb.B6 (n = 6) and Fvb (n = 6) mice at 4 and 7 weeks old. **c** The data represents the mean percentage of Ki67 positive cells ± SEM in the hyperplastic lesions of Fvb.B6 (9 weeks) and Fvb (5 weeks) mice (n = 5). **d** Ki67 immunostaining of mammary gland sections from Fvb.B6 (9 weeks) and Fvb (5 weeks) mice. **e** Kaplan–Meier curves showing the overall survival of Fvb.B6 (n = 31) and Fvb (n = 33) mice. Data represents the mean ± SEM, *P < 0.05, **P < 0.01, and ***P < 0.001

The Fvb mouse strain is susceptible to mammary tumors, while the B6 strain is more resistant [22]. To investigate the mechanisms underlying breast cancer initiation, we generated female MMTV-PyMT Fvb.B6 F1 hybrids with an MMTV-PyMT Fvb background. To determine whether the B6 genetic background affected the development of multifocal dysplastic lesions, we collected the right fourth (inguinal) mammary glands from the female MMTV-PyMT (Fvb) and MMTV-PyMT (Fvb.B6) mice at 4 weeks and 7 weeks old, respectively. The tissues were fixed in Carnoy’s solution and stained with carmine dye for two days. The area of hyperplasia in the mammary fat pads of the MMTV-PyMT (Fvb.B6) mice decreased 9.2-fold at 4 weeks and 5.6 fold at 7 weeks (Fig. 1a, b), suggesting that the B6 genetic background suppressed mammary tumor initiation. Compared with the Fvb mice, cellular proliferation was significantly reduced in the PyMT-induced hyperplastic lesions of the Fvb.B6 mice (Fig. 1c, d). Consequently, the Fvb.B6 mice bearing mammary tumors had a significantly prolonged overall survival (Fig. 1e).

### Primary tumor proliferation is suppressed by a B6 genetic background

**Fig. 2:**
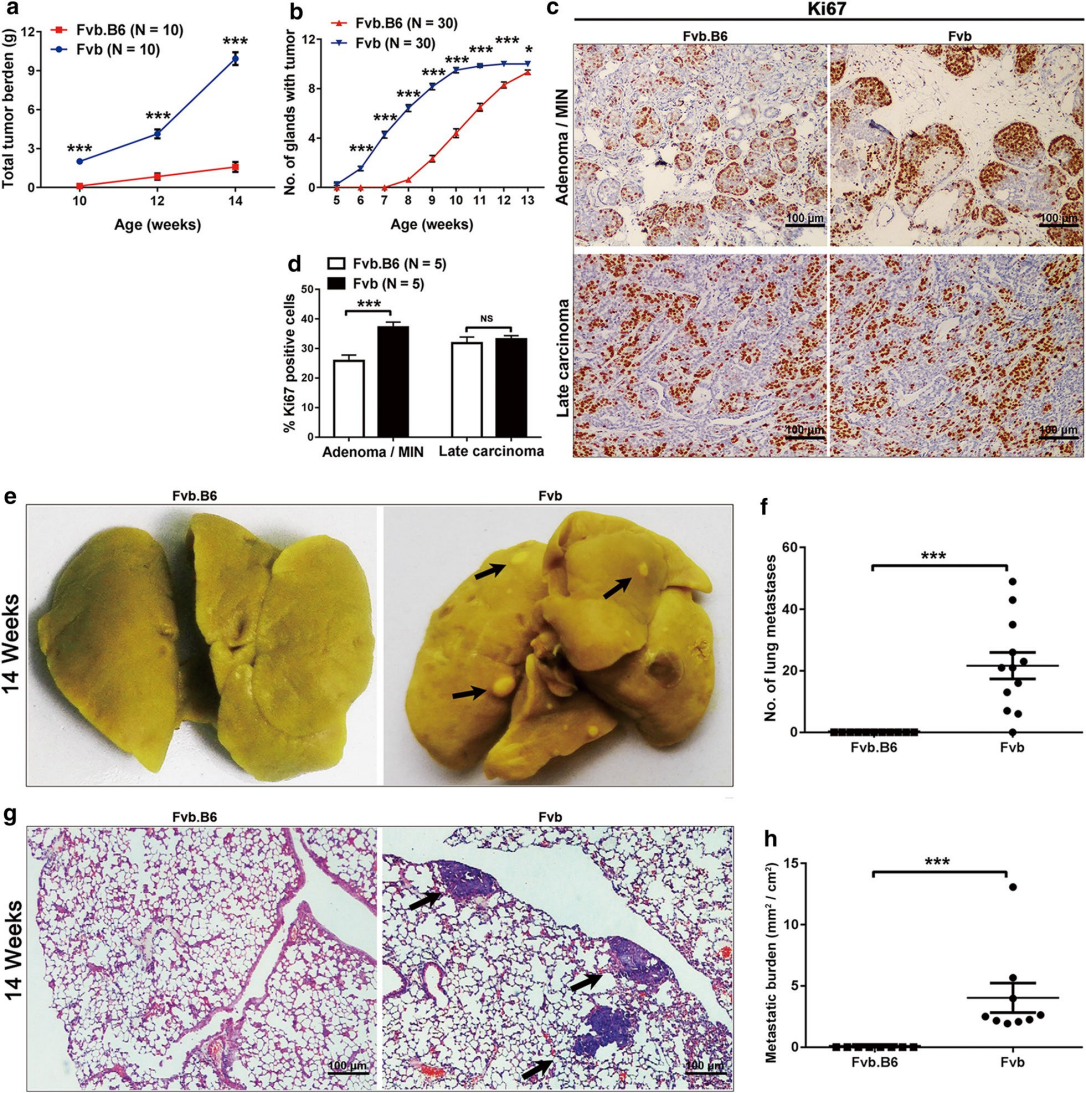
Mammary tumor growth and lung metastasis are suppressed by a B6 genetic background. **a** Total tumor burdens were determined in Fvb.B6 (n = 10) and Fvb (n = 10) mice by dissecting and weighing all tumors at 10, 12 and 14 weeks old. **b** The appearance of mammary tumors was monitored in Fvb.B6 (n = 30) and Fvb (n = 30) mice by weekly palpation and is represented on a Kaplan–Meier curve. **c** Ki67 staining of the mammary tumor sections from adenoma and late carcinoma. **d**. The mean percentage of Ki67 positive cells ± SEM in tumors from 5 mice/genotypes. **e**, **f** Bouin’s fixed lungs from Fvb.B6 and Fvb **e** mice were stained with Bouin’s solution to identify metastases (arrows). The data represents the mean total number of metastases ± SEM from Fvb.B6 (n = 9) and Fvb (n = 12) mice at 14 weeks old (**f**). **g**, **h** Formalin-fixed, paraffin-embedded lung sections from Fvb.B6 and Fvb **g** mice were stained with hematoxylin and eosin (H&E) to identify metastases (arrows). The data represents the mean total number of metastases from five lung sections/mouse ± SEM from Fvb.B6 (n = 8) and Fvb (n = 9) mice (**h**). Data represents the mean ± SEM, NS = non-significant, *P < 0.05, **P < 0.01, and ***P < 0.001

To assess the effects of B6′s background on mammary tumor progression, total tumor burden in the MMTV-PyMT (Fvb) and MMTV-PyMT (Fvb.B6) mice were measured at 10, 12 and 14 weeks old. The total tumor burden reduced significantly in the MMTV-PyMT (Fvb.B6) relative to the MMTV-PyMT (Fvb) breast tumors (Fig. 2a) and it was even lower in the MMTV-PyMT (Fvb.B6) mice at 14 weeks old compared with the MMTV-PyMT (Fvb) at 10 weeks, suggesting that the tumor growth in the MMTV-PyMT (Fvb.B6) mice was delayed for at least two weeks. Compared with the Fvb mice, tumor multiplicity was also significantly reduced in the Fvb.B6 mice, about 4 mammary glands of the Fvb mice had palpable tumors at 7 weeks versus 10 weeks in the Fvb.B6 mice (Fig. 2b). Consistently, a significant elevated proliferation rate measured by Ki67 positivity was found in the tumors of the Fvb mice in comparison with that of the Fvb.B6 mice at the adenoma/MIN stage (Fig. 2c, d).

### Lung metastasis is attenuated by a B6 background

The MMTV-PyMT mouse model develops lung metastases at a high frequency [18]. To determine the influence of the B6 background on metastasis, MMTV-PyMT (Fvb) and MMTV-PyMT (Fvb.B6) mice were euthanized at 14 weeks. The lungs were isolated and fixed in Bouin’s solution, and the metastases were quantified. The number of lung tumor nodules per mouse decreased significantly in the MMTV-PyMT (Fvb.B6) mice. Additionally, 100% of the MMTV-PyMT (Fvb) mice possessed pulmonary metastatic nodules compared with only 8.3% of the MMTV-PyMT (Fvb.B6) mice (Fig. 2e, f). HE staining showed that the number of lung metastases was also significantly lower in the MMTV-PyMT (Fvb.B6) mice compared with the MMTV-PyMT (Fvb) mice (Fig. 2g, h), suggesting that lung metastasis in the MMTV-PyMT (Fvb.B6) mice was significantly attenuated.

### Differentially expressed genes are enriched in immune-related pathways

**Fig. 3:**
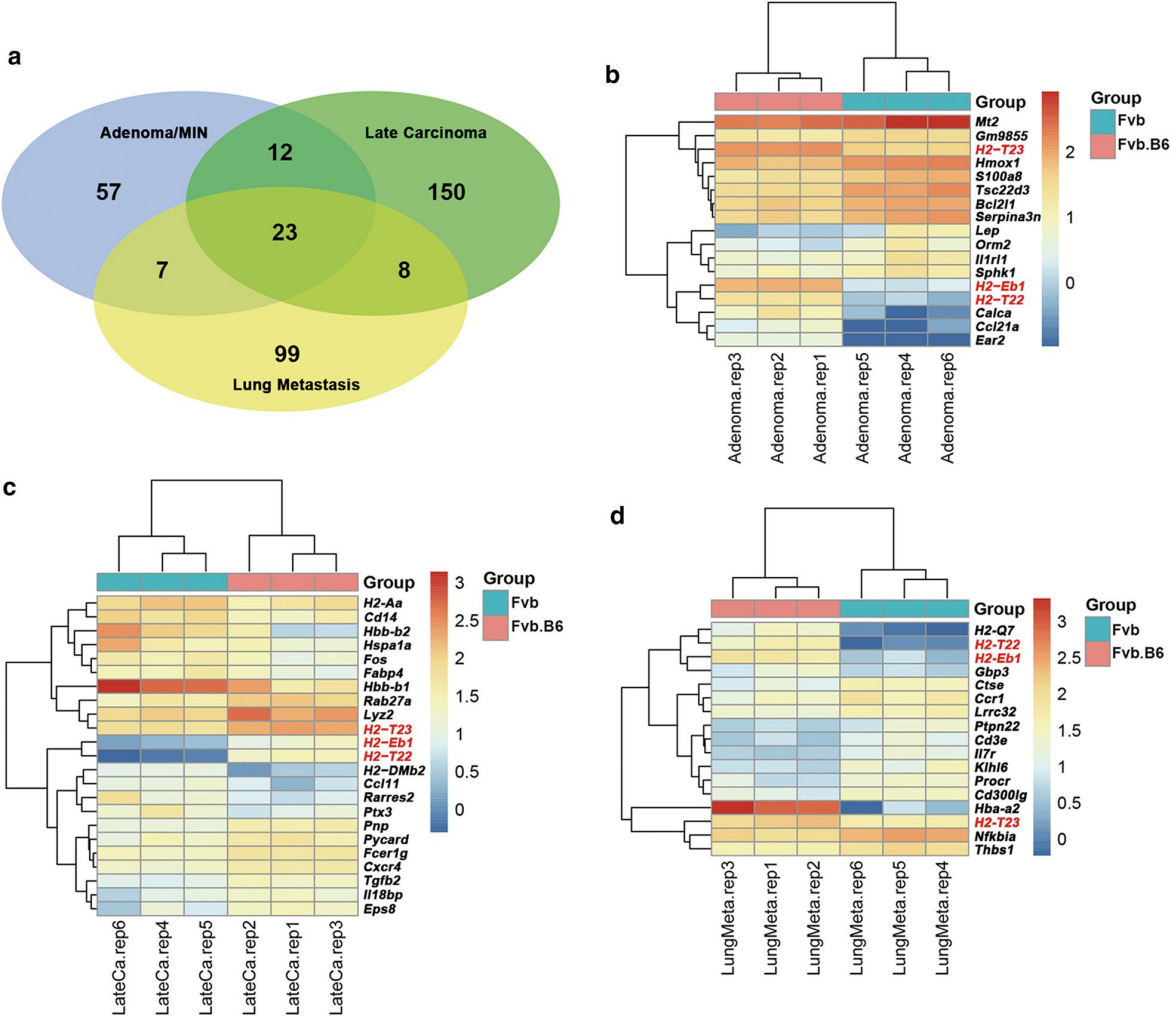
Differentially expressed genes in tumor progression and metastasis. **a** Venn diagram of DEGs. Genes involved in the antigen processing and presentation (GO: 19882), antigen processing and presentation of peptide antigen (GO: 48002) and the antigen processing and presentation of peptide antigen via MHC class I (GO: 02474) were collected as immune-related genes in this study. All DEGs in each tumor stage were used in depicting the Venn diagram. **b**–**d** Hierarchical clustering of immune-related DEGs. The expression pattern for the immune-related DEGs from adenoma **b**, late carcinoma of the mammary gland (**c**), and lung metastasis (**d**) are illustrated by the heatmap. Expression levels are depicted by the color bar beside the heatmap

We examined differentially expressed genes (DEGs) at each tumor stage. We compared three MMTV-PyMT (Fvb.B6) tumor samples with three MMTV-PyMT (Fvb) tumors, and identified 99, 193, and 137 DEGs at the adenoma stage, late adenocarcinoma stage, and lung metastasis, respectively (fold change > 2, FDR < 0.05, Additional file 2: Table S1; Additional file 3: Table S2; Additional file 4: Table S3). Among all 357 DEGs, a small proportion (6.4%, 23 genes) appeared at all three stages (Fig. 3a). Only three immune-related genes (*H2-Eb1, H2-T22 and H2-T23*), which were involved in the MHC-related immunity pathway appeared at all three stages (Fig. 3b–d).

**Fig. 4:**
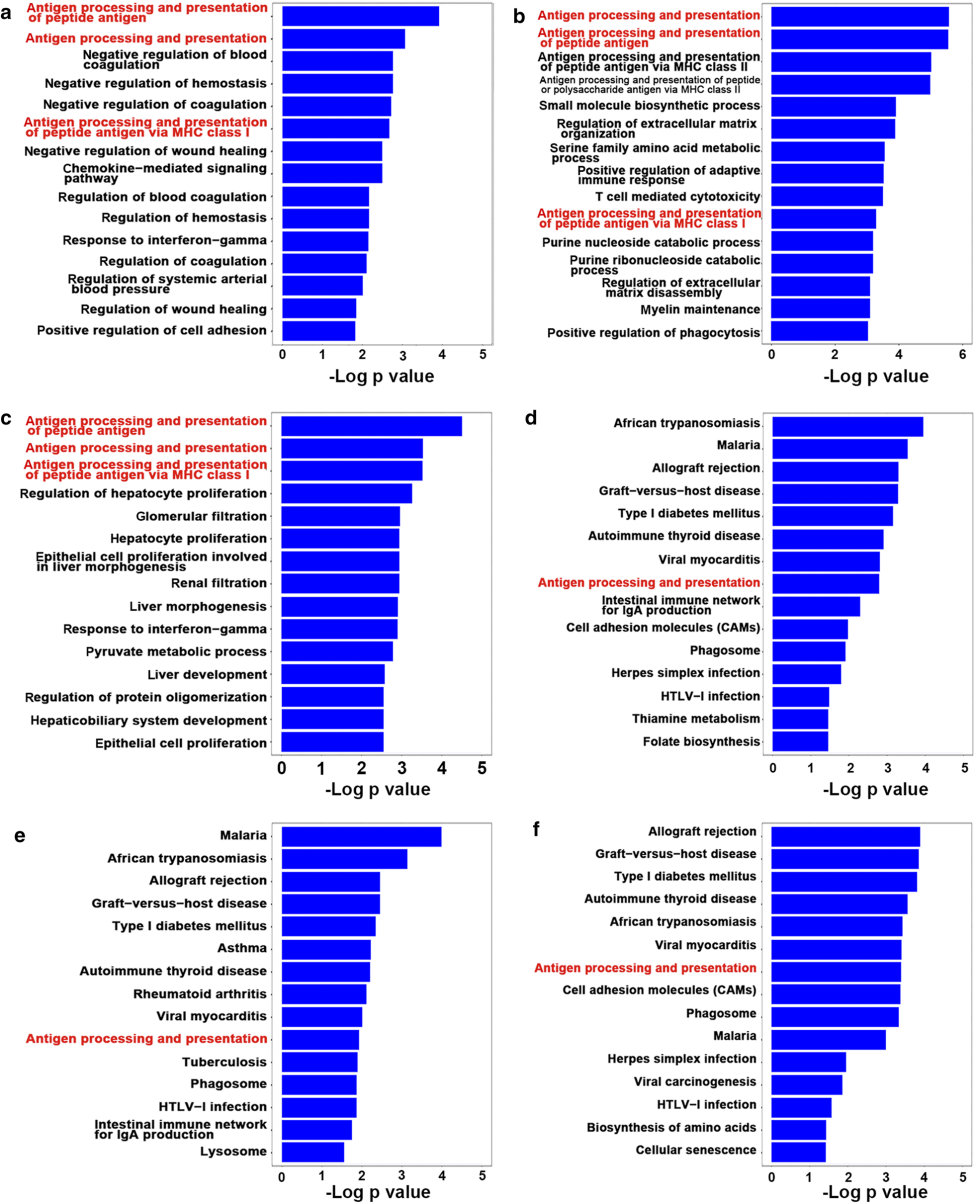
Gene enrichment analyses reveal the heavy involvement of immune function related genes. **a**–**c** Using the genomic expression data from the mouse model tumors, GO enrichment analyses of the up-regulated DEGs at three stages of breast cancer development were performed. The top 15 terms with the most significant p-value at adenoma (**a**), late carcinoma of the mammary gland (**b**), and lung metastasis (**c**) are plotted. **d**–**f** KEGG enrichment of the up-regulated DEGs at three stages. The top 15 terms with the most significant p-value at adenoma (**d**), late carcinoma (**e**), and lung metastasis (**f**) are plotted. Color gradient ranging from red to blue corresponds to the order of increasing p-values. All DEGs in each tumor stage were used for gene enrichment analyses, GO annotations of biological processes and the KEGG pathway were analyzed using the R package clusterProfiler v3.4.4

We then analyzed the GO and KEGG pathways of the DEGs identified at each stage. The antigen processing and presentation (GO: 19882) and the antigen processing and presentation of the peptide antigen (GO: 48002) were the top two enriched GO terms from a ranked list of the up-regulated DEGs at all three stages, and the antigen processing and presentation of the peptide antigen via MHC class I (GO: 02474) was in the top 10 enriched GO terms based on its p-value (Fig. 4a–c). Leukocyte migration (GO: 50900) was in the top 15 enriched GO terms in the down-regulated DEGs at all three stages or sites (Additional file 5: Fig. S2A–C). The antigen processing and presentation (KEGG: mmu04612) and allograft rejection pathways (KEGG: mmu05330) were found in the up-regulated DEGs (Fig. 4d–f). The difference in PyMT-induced breast cancer initiation, progression and metastasis between the F1 hybrids and Fvb background mice may be attributed to immunity, as there were many immune-related GO terms or pathways enriched in the top 15 at all three stages (Fig. 4). We picked out the immune-related genes in the DEGs identified at each stage (Fig. 3a–c). We were most interested in the antigen processing and presentation pathway because it was significantly enriched in the up-regulated DEGs by both the GO enrichment and KEGG pathway analysis, and it is the pathway involved when antigen-presenting cells express antigen (peptide or lipid) on its cell surface in association with an MHC protein complex [23].

**Fig. 5:**
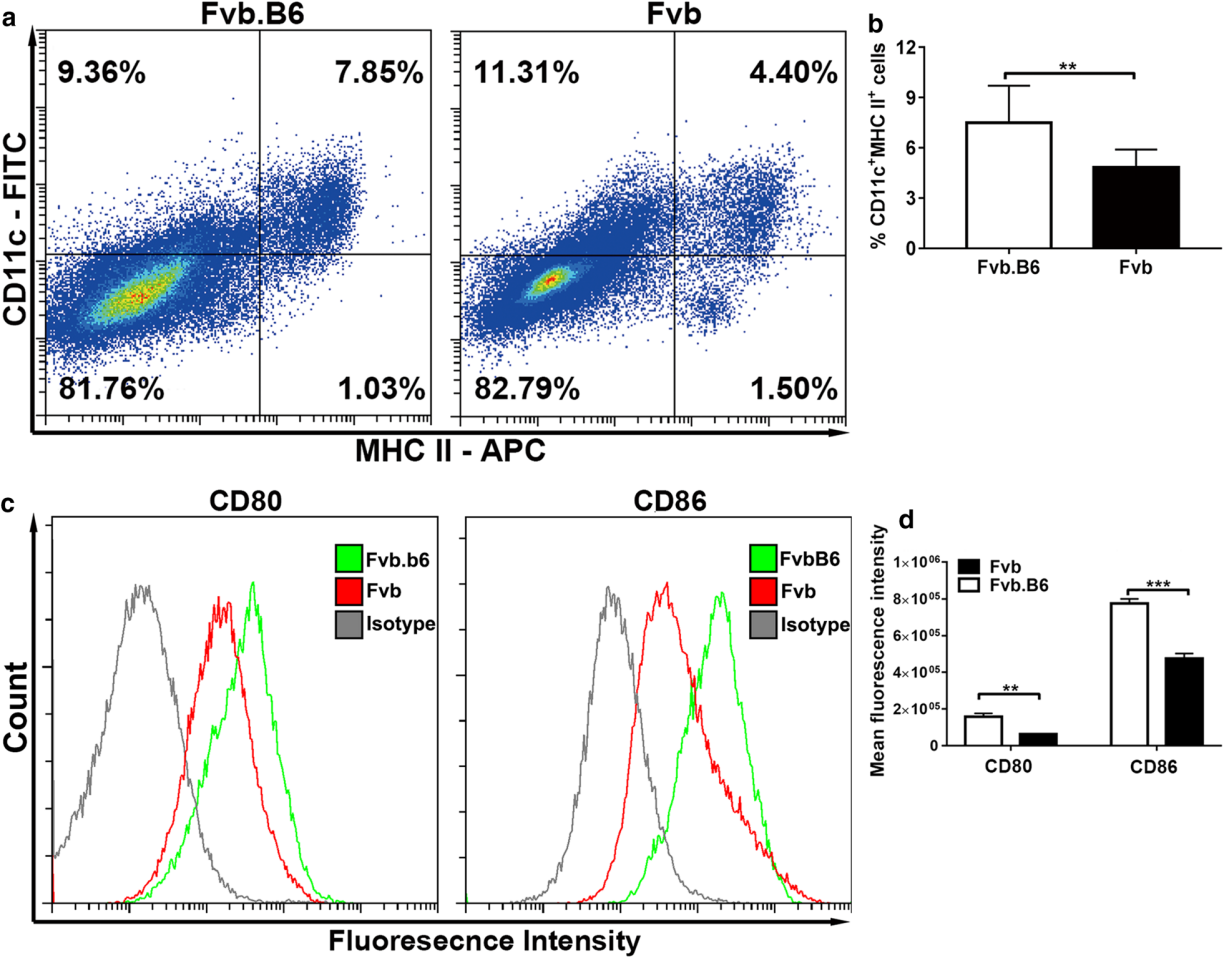
More tumor infiltrating dendritic cells were present in Fvb.B6 mice. **a**, **b** Total tumor infiltrating CD11c + MHC-II + DC from Fvb.B6 and Fvb mice were analyzed by flow cytometry analysis. Representative plots depicting total tumor infiltrating CD11c + MHC-II + DC from Fvb.B6 mice and Fvb mice at the early carcinoma stage (**a**). The percentage of CD11c + MHC II + DC from Fvb.B6 (n = 10) and Fvb mice (n = 10) were pooled, the data represents the mean ± SEM (**b**). **c** Representative plots depicting CD80 and CD86 expression by CD11c + MHC II + DC from Fvb.B6 and Fvb mice at the early carcinoma stage. **d** CD80 and CD86 expression by CD11c + MHC II + DC from Fvb.B6 (n = 10) and Fvb mice (n = 10) with means ± SEM shown. *, p < 0.05; **, p < 0.01 by Student’s t-test

To validate these antigen-presenting related genes and cells, we found that five MHC I coding genes (H2-T22, H2-T23, H2-K1, H2-Q9 and H2-Q10) and five MHC II coding genes (H2-Eb1, H2-Aa, H2-Ab1, H1-DMb1 and H2-DMa) were expressed at significantly higher levels in breast tumor tissues in the Fvb.B6 mice than in the Fvb mice (Additional file 6: Fig. S3). This suggests that Fvb.B6 mice had better antigen processing and presentation activity. DCs are the main APCs capable of inducing immunity to foreign antigens [24]. We found that the Fvb.B6 mice had a higher proportion of tumor infiltrating dendritic cells than the Fvb mice (Fig. 5a, b). The surface expression of CD80 and CD86 on DCs also increased in the Fvb.B6 mice (Fig. 5c, d), implying that the Fvb.B6 mice have more mature DCs compared with the Fvb mice.

### IL-6 inhibits the maturation of dendritic cells in Fvb mice, and VEGF level is elevated in Fvb mice

**Fig. 6:**
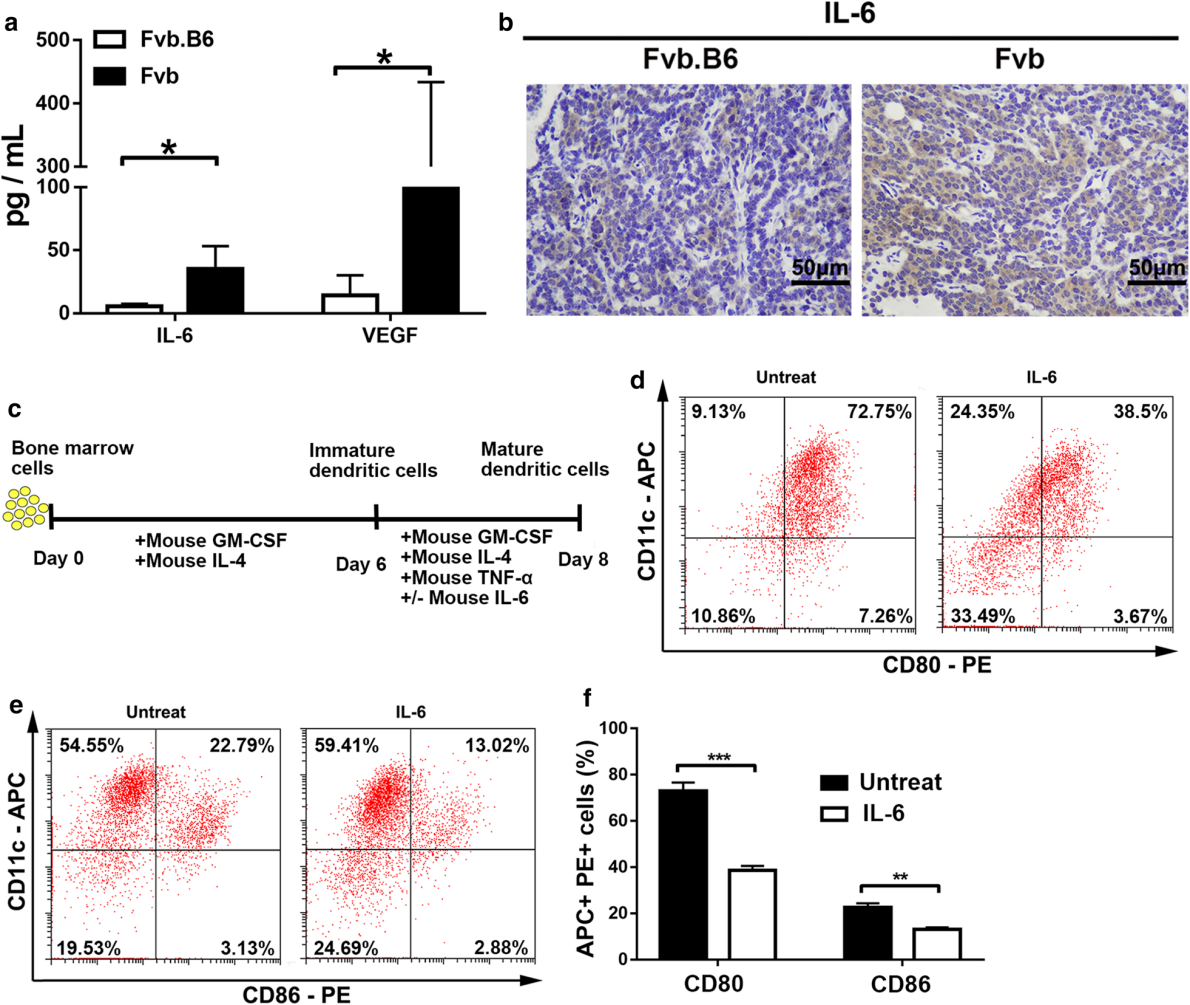
IL-6 suppresses the maturation of dendritic cells in Fvb mice, and the VEGF level is decreased in Fvb.B6 mice. **a** Level of serum IL-6 and VEGF in Fvb.B6 (n = 5) and Fvb (n = 5) mice was detected by a Mcytomag-70 K-32 Mouse Cytokine/Chemokine Magnetic Bead Panel. **b** IHC staining of IL-6 in breast cancer tissues from Fvb.B6 (n = 10) and Fvb (n = 10) mice at adenoma. **c** Experimental strategy and BMDCs were induced from day 6 to day 8, with or without 10 ng/mL of mouse IL-6 treatment on day 6. **d**, **e** Plots depicting CD80 (**d**) and CD86 (**e**) expression by BMDCs after 2 days of coculturing with or without IL-6. **f** CD80 and CD86 expression by BMDCs (n = 5) with means ± SEM shown

Many tumor-derived soluble mediators are able to impair DCs’ maturation and function in certain types of cancer [11]. To investigate the factors influencing DC maturation, we measured the plasma chemokine levels in Fvb and Fvb.B6 mice using a multiplex MCYTOMAG-70K-32 assay, and found that the plasma levels of IL-6 and VEGF were significantly reduced in Fvb.B6 mice (Fig. 6a). What's more, the expression level of IL-6 was also decreased in the breast tumor tissues of the Fvb.B6 mice than in the Fvb mice (Fig. 6b). To determine the influence of IL-6 on DCs’ maturation, we established an in-vitro culture system to generate BMDCs (Fig. 6c). After 2 days of coculturing with IL-6, we found that the surface expression of CD80 and CD86 on BMDCs were significantly decreased upon IL-6 treatment (Fig. 6d–f), suggesting that IL-6 suppressed the maturation of DCs in Fvb mice.

## Discussion

There are tremendous clinical demands to postpone the onset of cancer, especially in patients with an inherited susceptibility early in their lives. Given the same genetic defects that drive breast cancer formation, the onset of breast cancer among certain populations differs greatly in their life spans, with the underlying mechanism to be determined. In our mouse models, PyMT-induced mammary tumor initiation was significantly delayed and cancer progression and metastasis were significantly suppressed in Fvb.B6 hybrid mice with the introduction of a B6 genetic background. We demonstrated, for the first time, that transcriptional profiling is associated with distinct stages in mammary tumor progression and metastasis. We also observed that temporal global mRNA levels are differentially expressed in the two different genetic backgrounds of the PyMT mice. The GO enrichment and KEGG pathway analysis showed that the immune-related GO terms or pathways were the main difference between the two types of mice with different genetic backgrounds. Previous studies have shown that B6 mice have a H-2b haplotype that results in immunologically mediated resistance to polyoma virus infection and its tumorigenesis [9]. Whereas, Fvb mice have a H-2q haplotype, which may be an additional MHC locus that confers susceptibility to polyoma virus-induced tumorigenesis. Consistent with these preliminary observations, the antigen processing and presentation pathway (GO: 19882, KEGG: mmu04612) was screened out at all three stages by the two enrichment methods in our study.

DCs are professional antigen presenting cells (APCs), and their main function is to process antigen material and present it to the T cells [25]. In our study, flow cytometry analysis showed that the Fvb.B6 mice had a higher proportion of dendritic cells than the Fvb mice (Fig. 5), suggesting higher levels of antitumor immunity existed in the Fvb.B6 mice. A previous study showed that IL-6 conversely inhibited the expression of CD80 and CD86 in DCs [26], and IL-6 significantly inhibited the maturation of DCs via activation of the JAK2/STAT3 pathway [27]. Our study showed consistent results, the IL-6 level was significantly higher in the plasma and breast tumor tissues of the Fvb mice, and IL-6 also impaired the maturation of the BMDCs in the Fvb mice in vitro (Fig. 6). VEGF is another well-known inhibitor for DC function, and we also revealed its reduced expression in Fvb.B6 mice.

## Conclusions

**Fig. 7:**
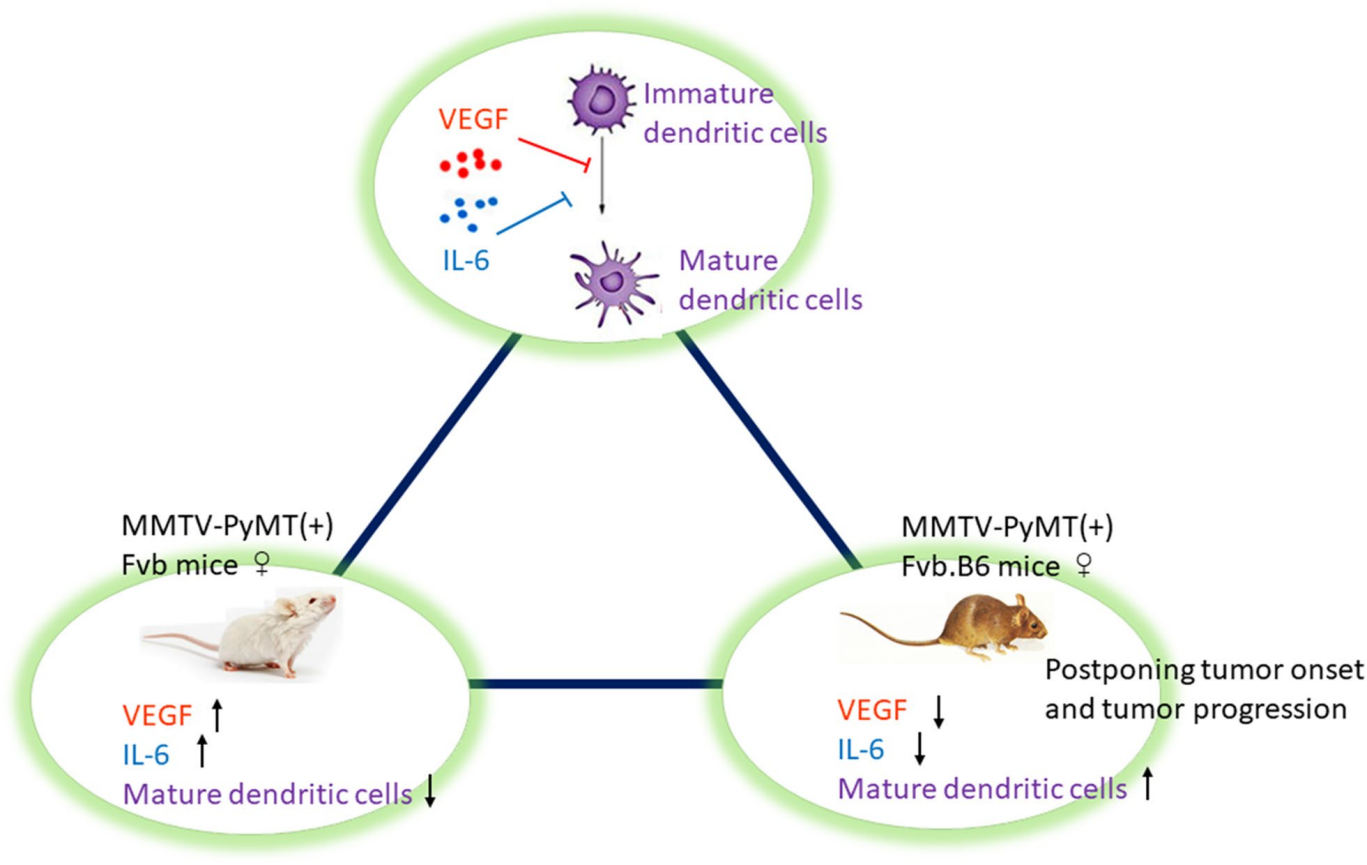
Schematic presentation of mechanism underlying VEGF and IL-6 mediated dendritic cells. Upregulated VEGF and IL-6 could prevent the process of DCs’ maturation in Fvb mice compared with Fvb.B6 mice. High ratios of dendritic cells in Fvb.B6 mice would partially postpone tumor onset and tumor progression

PyMT-induced mammary tumor initiation, progression and metastasis can be significantly suppressed in Fvb.B6 mice compared with Fvb mice. The enhanced immunity in Fvb.B6 mice displayed a higher proportion of matured dendritic cells, which play an essential role in delaying the onset of cancer and suppressing cancer progression. Fvb.B6 mice had a higher proportion of matured dendritic cells than Fvb mice. Elevated IL-6 and VEGF levels in Fvb mice may impair the maturation of DCs (Fig. 7). Taken together, our findings suggest that in a murine breast cancer model, alterations of local immunity in the tumor microenvironment can postpone tumor onset and delay tumor progression.

## Supplementary Information

**Additional file 1: Fig. S1.** The experiment flow chart of this study was depicted. Fvb.B6 F1 female (brown color) hybrids were generated by crossing FVB/N-Tg(MMTV-PyMT)634Mul male (white color) mice to C57BL/6J female (black color) mice (Fig. S1A), Mammary tumors were harvested when the diameter of the largest tumor per mouse reached approximately 2 millimeters and 10 millimeters, which correspond to the Adenoma/MIN and late carcinoma stages. Lung metastases were harvested at the age of 13 weeks for Fvb mice and 16 weeks for F1 hybrids (Fig. S1B).

**Additional file 2: Table S1.** Diffentially expressed genes of the adenoma/MIN stage.

**Additional file 3: Table S2.** Diffentially expressed genes of the late carcinoma stage.

**Additional file 4: Table S3.** Diffentially expressed genes of the lung metastasis.

**Additional file 5: Fig. S2.** GO enrichment of down-regulated DEGs at three stages. The top 15 terms with the most significant p values at adenoma stage (Fig. S2A), late adenocarcinoma stage (Fig. S2B), and lung metastasis (Fig. S2C) are plotted. Color gradient ranging from red to blue corresponds to increasing p-values.

**Additional file 6: Fig. S3.** qPCR verification and validation of gene expression. qPCR validation using RNAs from independent samples from adenoma stage. Data represent the mean ± SEM, n=6; ***P < 0.001 and ****P < 0.0001.

## Data Availability

The authenticity of this article has been validated by uploading the key raw data onto the Research Data Deposit public platform (www.researchdata.org.cn), with the approval RDD number: RDDB2020001031.

## Abbreviations

MMTV-PyMT: Mouse Mammary Tumor Virus Polyoma Middle T Antigen
Fvb: FVB/NJ
B6: C57BL/6J
DEGs: Differentially expressed genes
GO: Gene ontology
KEGG: Kyoto Encyclopedia of Genes and Genomes
EtOH: Absolute ethanol
FPKM: Fragments Per Kilobase of transcript per Million fragments mapped
