# Supplementary figures and images for: Postponing tumor onset and tumor progression can be achieved by alteration of local tumor immunity
Source: Cancer Cell Int. 2021 Feb 10;21:97. doi: 10.1186/s12935-021-01765-7 (PMC7874464; doi:10.1186/s12935-021-01765-7)

## Slide 1
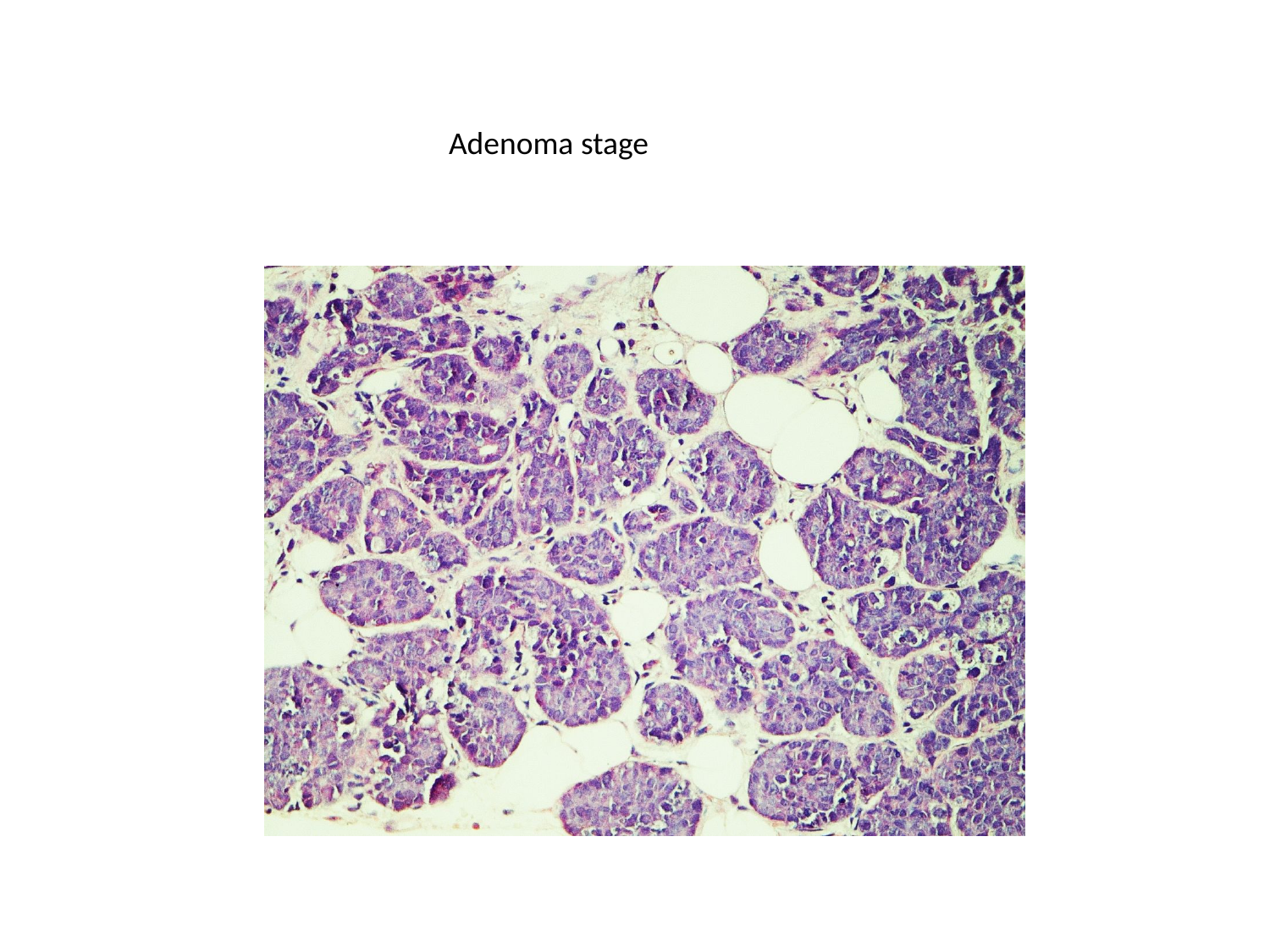

Adenoma stage

## Slide 2
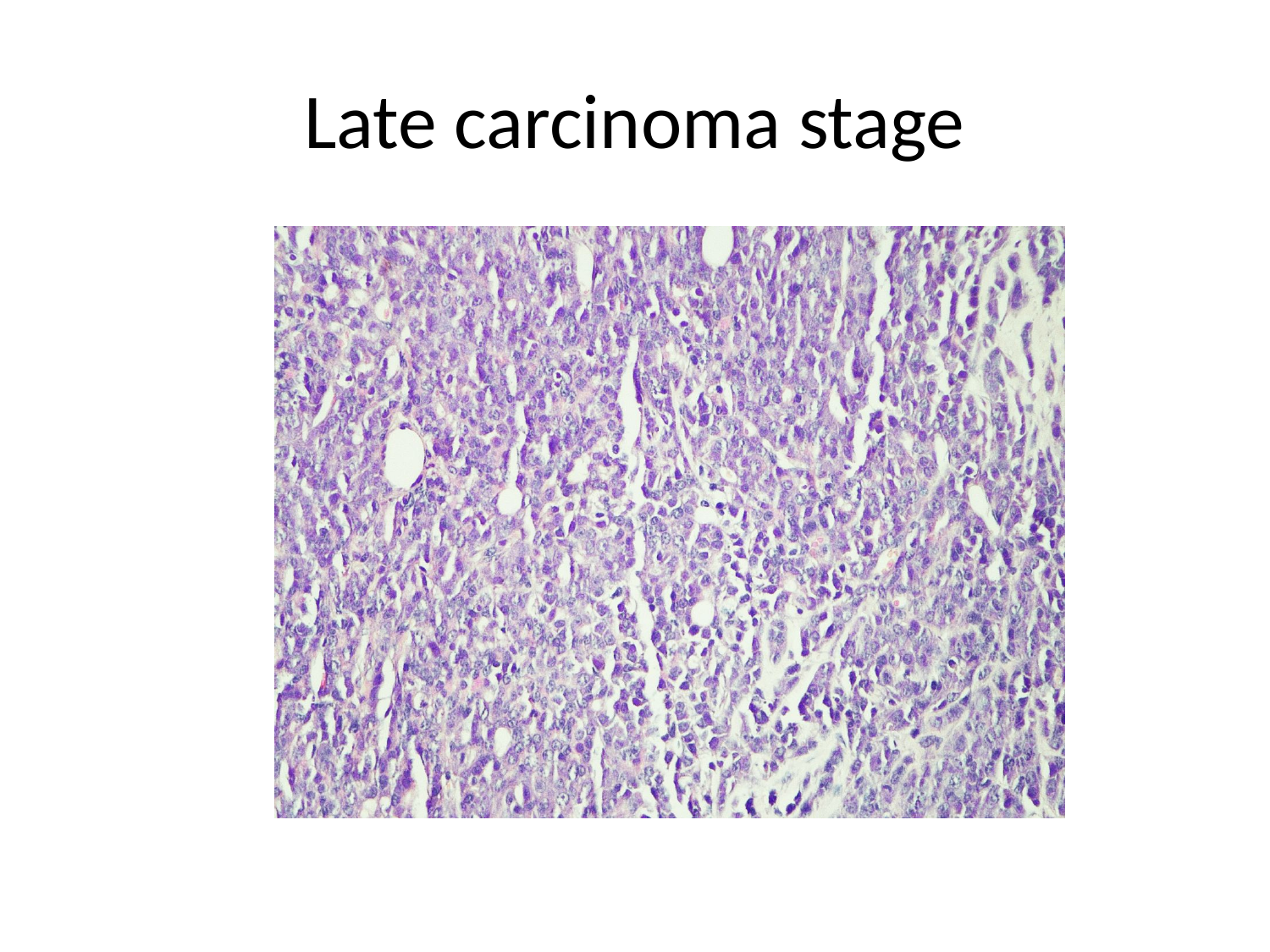

# Late carcinoma stage

## Slide 3
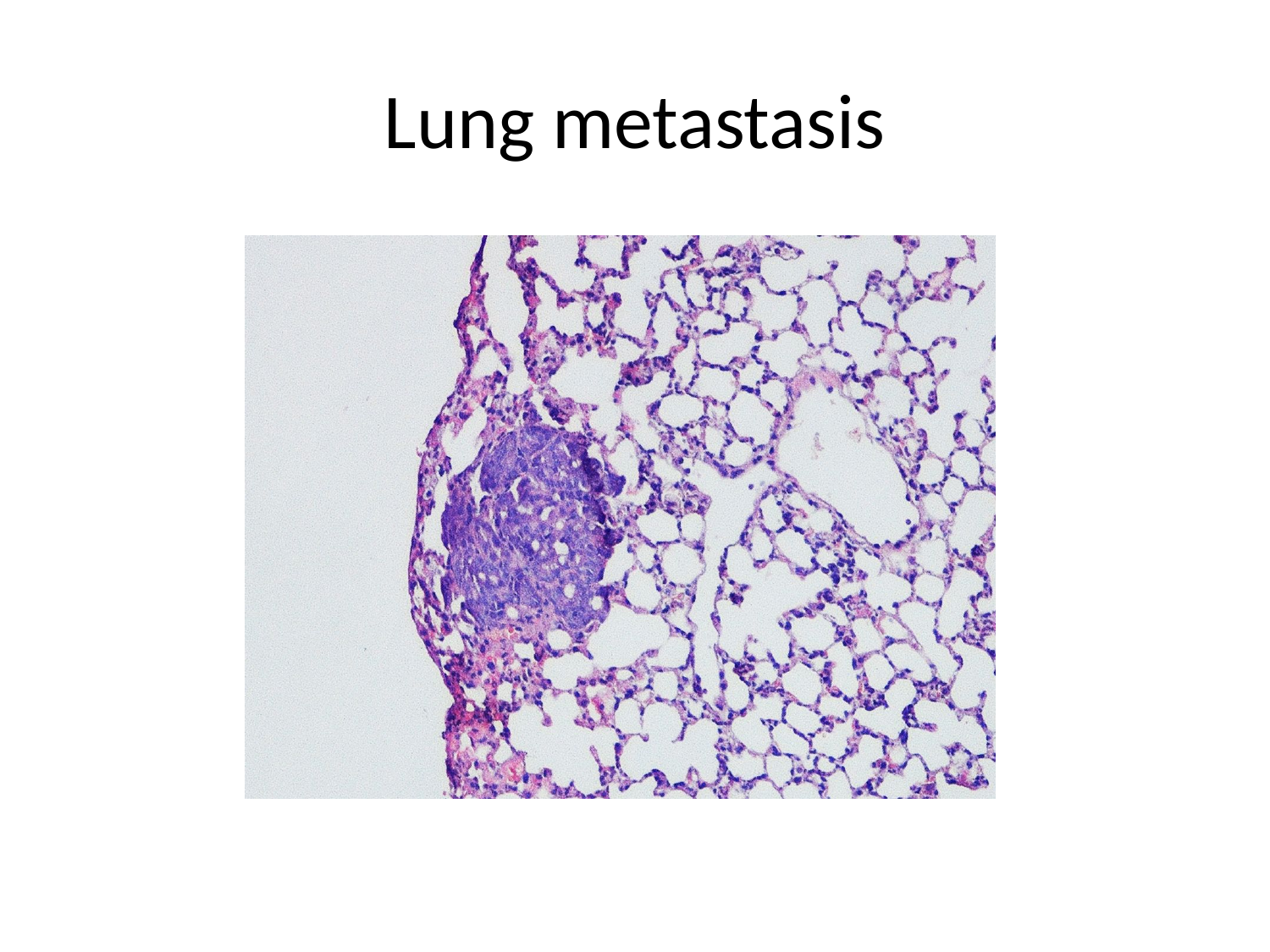

# Lung metastasis

Supplement: Supplementary file 1 — Additional file 1: Fig. S1. The experiment flow chart of this study was depicted. Fvb.B6 F1 female (brown color) hybrids were generated by crossing FVB/N-Tg(MMTV-PyMT)634Mul male (white color) mice to C57BL/6J female (black color) mice (Fig. S1A), Mammary tumors were harvested when the diameter of the largest tumor per mouse reached approximately 2 millimeters and 10 millimeters, which correspond to the Adenoma/MIN and late carcinoma stages. Lung metastases were harvested at the age of 13 weeks for Fvb mice and 16 weeks for F1 hybrids (Fig. S1B). [file 12935_2021_1765_MOESM1_ESM.pptx]

## Slide 1
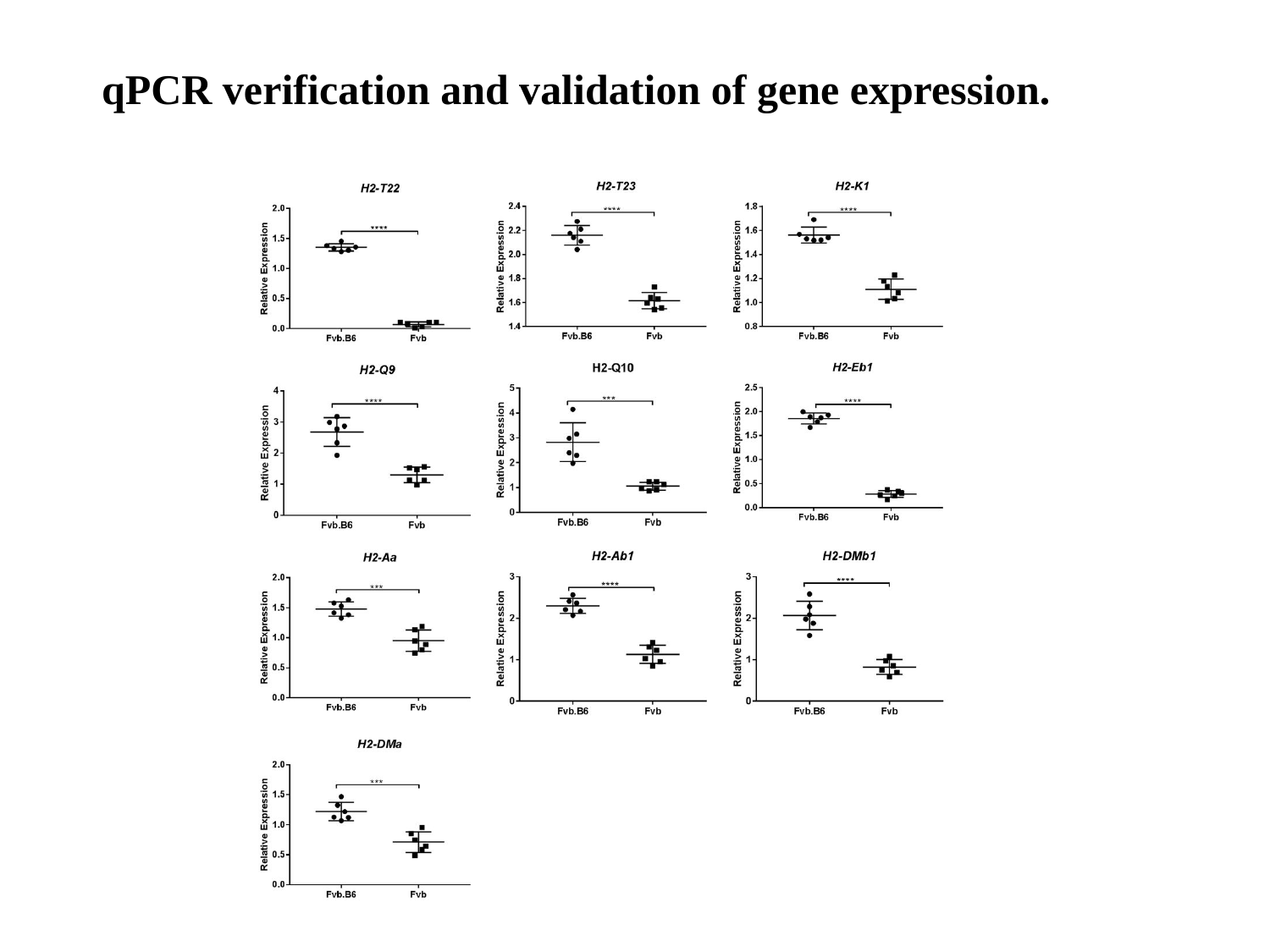

qPCR verification and validation of gene expression.

Supplement: Supplementary file 6 — Additional file 6: Fig. S3. qPCR verification and validation of gene expression. qPCR validation using RNAs from independent samples from adenoma stage. Data represent the mean ± SEM, n=6; ***P < 0.001 and ****P < 0.0001. [file 12935_2021_1765_MOESM6_ESM.pptx]
