# Supplementary material for: Postponing tumor onset and tumor progression can be achieved by alteration of local tumor immunity
Source: Cancer Cell Int. 2021 Feb 10;21:97. doi: 10.1186/s12935-021-01765-7 (PMC7874464; doi:10.1186/s12935-021-01765-7)
Supplement: Supplementary file 5 — Additional file 5: Fig. S2. GO enrichment of down-regulated DEGs at three stages. The top 15 terms with the most significant p values at adenoma stage (Fig. S2A), late adenocarcinoma stage (Fig. S2B), and lung metastasis (Fig. S2C) are plotted. Color gradient ranging from red to blue corresponds to increasing p-values. [file 12935_2021_1765_MOESM5_ESM.pptx]

## Slide 1
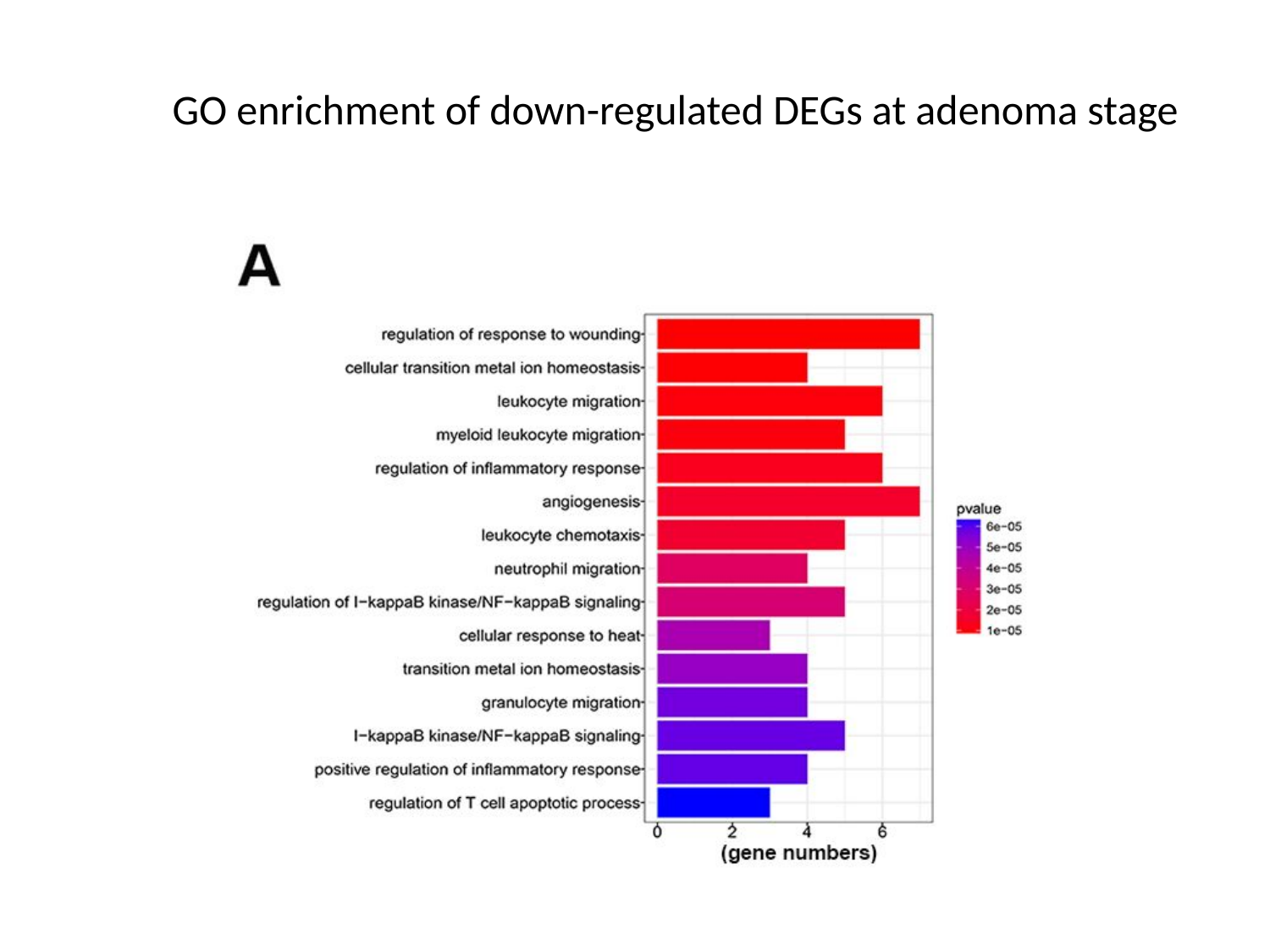

GO enrichment of down-regulated DEGs at adenoma stage

## Slide 2
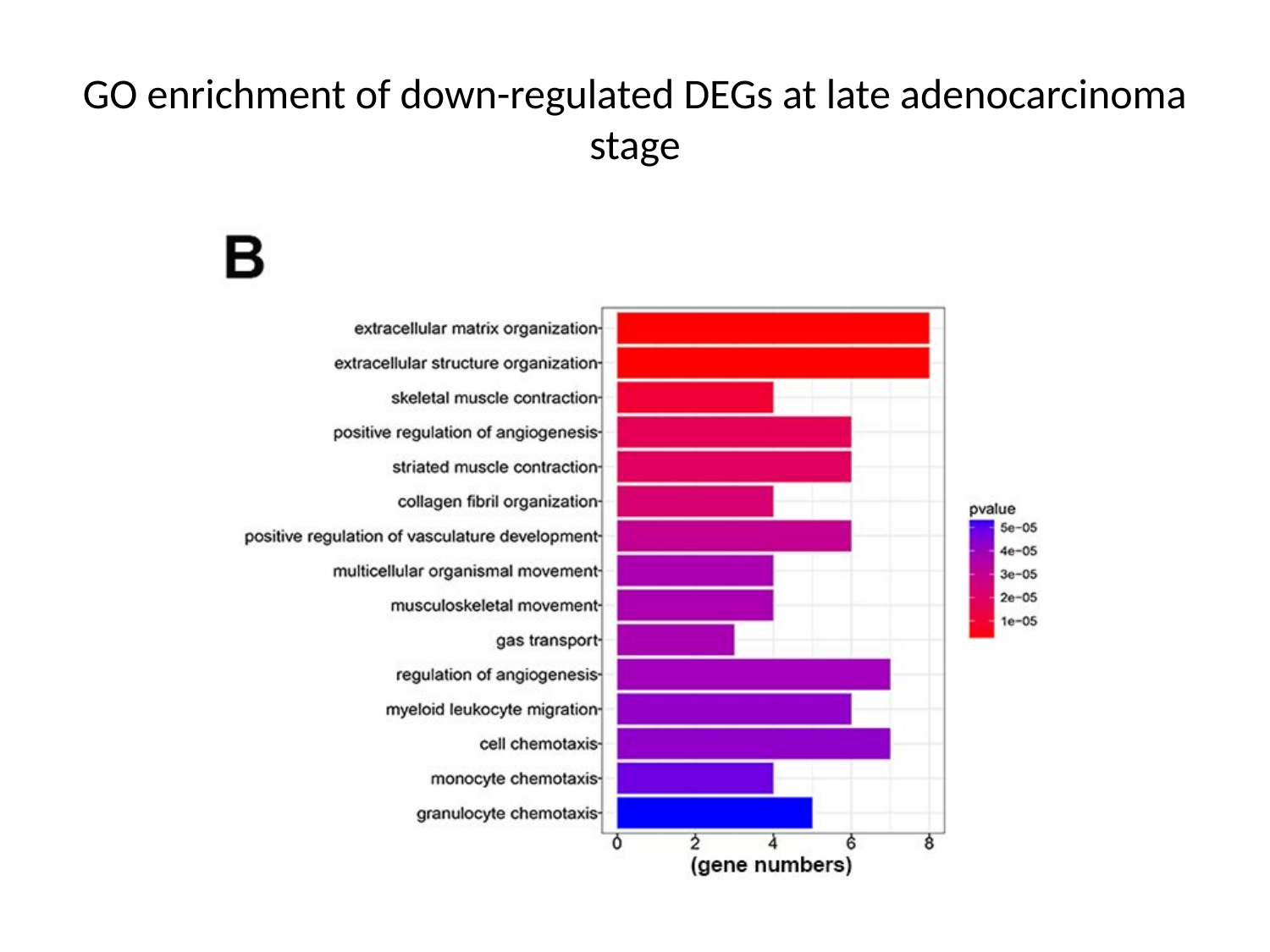

# GO enrichment of down-regulated DEGs at late adenocarcinoma stage

## Slide 3
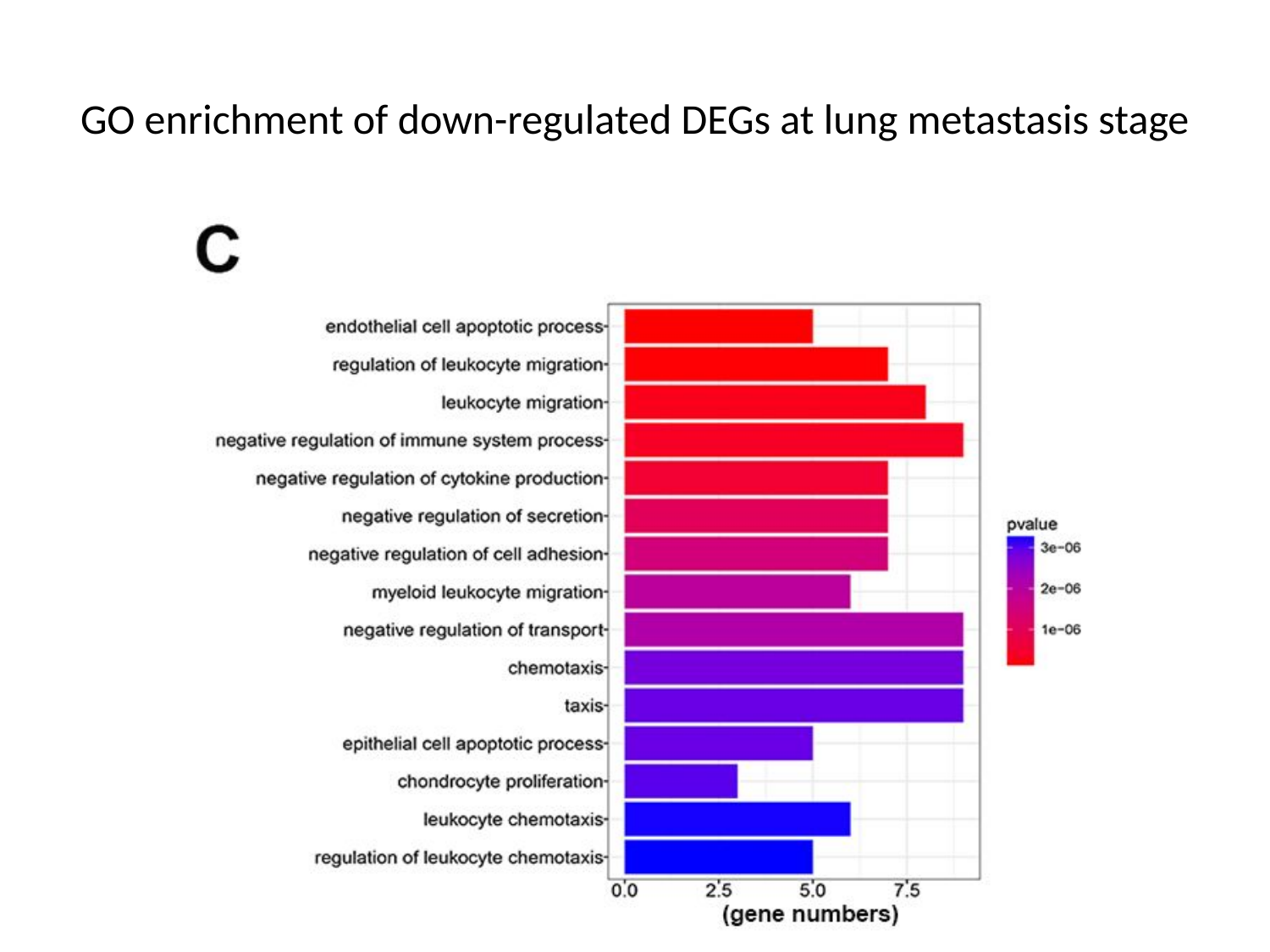

# GO enrichment of down-regulated DEGs at lung metastasis stage
